# Sleep, School Performance, and a School-Based Intervention among School-Aged Children: A Sleep Series Study in China

**DOI:** 10.1371/journal.pone.0067928

**Published:** 2013-07-10

**Authors:** Shenghui Li, Lester Arguelles, Fan Jiang, Wenjuan Chen, Xingming Jin, Chonghuai Yan, Ying Tian, Xiumei Hong, Ceng Qian, Jun Zhang, Xiaobin Wang, Xiaoming Shen

**Affiliations:** 1 MOE - Shanghai Key Laboratory of Children’s Environmental Health, Xinhua Hospital, Shanghai Jiao Tong University School of Medicine, Shanghai, P. R. China; 2 School of Public Health, Shanghai Jiao Tong University, Shanghai, P. R. China; 3 Mary Ann and J Milburn Smith Child Health Research Program, Northwestern University Feinberg School of Medicine and Children’s Memorial Hospital and Children’s Memorial Research Center, Chicago, Illinois, United States of America; 4 Shanghai Children’s Medical Center, School of medicine, Shanghai Jiao Tong University, Shanghai, P. R. China; 5 Center on Childhood Origins of Disease, Department of Population, Family and Reproductive Health, Johns Hopkins Bloomberg School of Public Health, Baltimore, Maryland, United States of America; National Taiwan University, College of Medicine, Taiwan

## Abstract

**Background:**

Sufficient sleep during childhood is essential to ensure a transition into a healthy adulthood. However, chronic sleep loss continues to increase worldwide. In this context, it is imperative to make sleep a high-priority and take action to promote sleep health among children. The present series of studies aimed to shed light on sleep patterns, on the longitudinal association of sleep with school performance, and on practical intervention strategy for Chinese school-aged children.

**Methods and Findings:**

A serial sleep researches, including a national cross-sectional survey, a prospective cohort study, and a school-based sleep intervention, were conducted in China from November 2005 through December 2009. The national cross-sectional survey was conducted in 8 cities and a random sample of 20,778 children aged 9.0±1.61 years participated in the survey. The five-year prospective cohort study included 612 children aged 6.8±0.31 years. The comparative cross-sectional study (baseline: n = 525, aged 10.80±0.41; post-intervention follow-up: n = 553, aged 10.81±0.33) was undertaken in 6 primary schools in Shanghai. A battery of parent and teacher reported questionnaires were used to collect information on children’s sleep behaviors, school performance, and sociodemographic characteristics. The mean sleep duration was 9.35±0.77 hours. The prevalence of daytime sleepiness was 64.4% (sometimes: 37.50%; frequently: 26.94%). Daytime sleepiness was significantly associated with impaired attention, learning motivation, and particularly, academic achievement. By contrast, short sleep duration only related to impaired academic achievement. After delaying school start time 30 minutes and 60 minutes, respectively, sleep duration correspondingly increased by 15.6 minutes and 22.8 minutes, respectively. Moreover, intervention significantly improved the sleep duration and daytime sleepiness.

**Conclusions:**

Insufficient sleep and daytime sleepiness commonly existed and positively associated with the impairment of school performance, especially academic achievement, among Chinese school-aged children. The effectiveness of delaying school staring time emphasized the benefits of optimal school schedule regulation to children’s sleep health.

## Introduction

For a variety of reasons, either by societal changes or due to lifestyle choice, chronic sleep loss is increasingly common in our hectic modern society [1]–[4]. Over the past century, sleep duration in children and adolescents showed a trend of 0.75 min less per year [2]. It has been estimated that 15%–75% of school-aged children are not getting sufficient sleep [5], [6]. It is well established that insufficient sleep can result in excessive daytime sleepiness and, therefore, links to problems with attention, concentration, impulsivity, mood regulation, and cognitive functioning [7]–[10]. Furthermore, more recent studies have revealed that sleep loss is implicated in the pathogenesis of childhood obesity [11]–[13].

In light of the increasing prevalence and negative consequences of sleep loss, promotion of healthy sleep habits and reform of public policies, such as school schedule modification, have been advocated to improve children’s sleep [14]–[16]. This need is further highlighted by the fact that, although the advances in sleep science are encouraging, awareness and appreciation of the importance of sleep among the general public and among health care professionals are still extremely limited [17]. Our previous study indicated that the most marked reduction in sleep duration among children occurs at the time they enter into primary school–a transition characterized by delayed bedtimes yet fixed school morning wake-up time [18], [19]. To that end, attention to sleep health should be focused on school-aged children, as well as the high-priority target population for sleep intervention.

Sleep in children is connected with sociocultural background characteristics [20]. As a Confucian country, China has a strong tradition of Confucianism and intrinsic sociocultural values, which differs greatly from Western countries. As a developing country, China varies from more developed countries in socioeconomic status. Moreover, China has a unique one-child policy. These characteristics may make sleep characteristics of Chinese children different from their peers in other countries. In Chinese society, particular emphases and special expectations are put on children’s academic performance, especially for the only child. It is becoming a common practice for children, from primary school or even earlier, to burden themselves with tremendous academic pressure and to spend more and more time on their studies even with the sacrifice of sleep time [19], [21]. It was shown that the mean school period in Chinese school-aged children was 9.67 hours [21]. In addition, our previous study demonstrated that 24.9% and 48.8% of school-aged children did homework after school for two or more hours per day during weekdays and on weekends, respectively [19]. A cross-cultural comparison of sleep habits between Chinese and American children showed that, compared with their American peers, Chinese school-aged children go to bed later, wake up earlier, and sleep nearly one hour less per day [20].

Over the past several years, accumulating studies indicated that sleep plays a particularly important role in attention, learning process, memory consolidation, and therefore, in children’s academic achievement and school performance [22], [23]. However, due to different methodologies and varied sample characteristics, it is difficult to draw general conclusions about the association of sleep with academic achievement and school performance [22], [23]. For example, a more recent meta-analysis demonstrated that, compared with sleep quality, sleep duration showed smaller and inconsistent significance with regard to school performance [23]. In addition, it is far from clear which aspects of school performance are most strongly related with sleep [22], [23]. In the context of Chinese society, where it is common for children to sacrifice sleep for better academic achievement, a full understanding of the relationship between sleep and school performance is greatly needed.

Based upon the above, a sleep series study was specially designed to obtain insight into sleep health among Chinese school-aged children by examining: 1) sleep patterns and their distribution with demographic, district, ethnicity, and socio-economic status in a large national cross-sectional survey; 2) the longitudinal associations of sleep duration and daytime sleepiness with school performance in a prospective cohort study; 3) the effectiveness of a school-based sleep intervention scheme using a comparative cross-sectional analysis of pre- and post-intervention surveys.

## Methods

### Ethics Statement

The ethical application and consent procedure of this study were approved by the Ministry of Education of the People’s Republic of China and Ethics Committee of Shanghai Jiaotong University School of Medicine.

### Study Protocol

The purposes of this research project were explained to the principals and teachers of the target schools. After the permissions were obtained from these schools, students who were eligible to participate in this study were invited to do anthropometric measurements. Meanwhile, a questionnaire was taken to their parents, with a letter explaining the objectives of the project and instructions on how to complete the questionnaire. Children and parents were told that participation in the survey was voluntary and the survey was anonymous. Only those children whose parents (caregivers) returned signed consent participated in the study. During the longitudinal study, the same serial number was used for the same child at baseline survey and follow-up survey.

### Study Design and Sample

**Figure 1:**
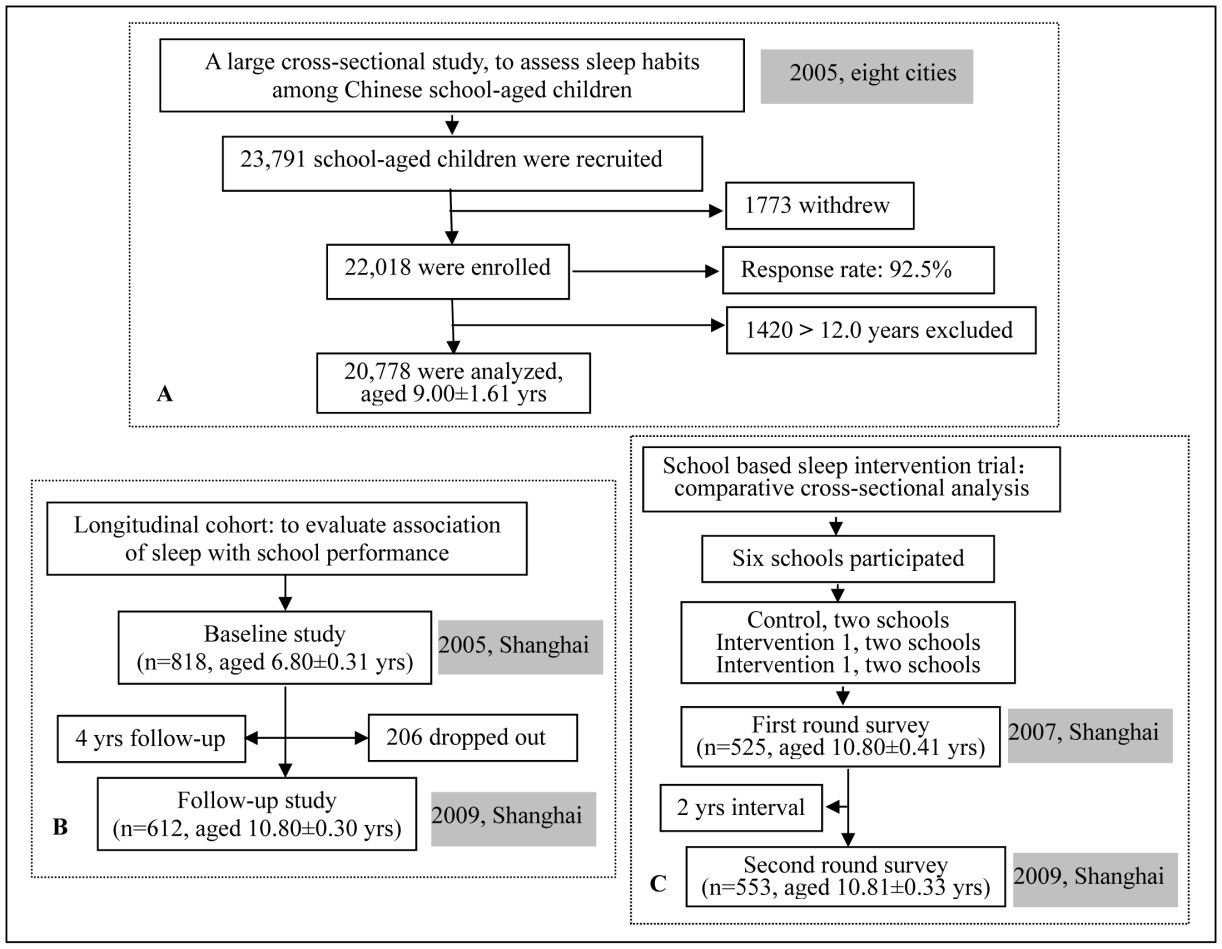
The study profile of sleep series study. A: Cross-sectional study. B: Longitudinal cohort study. C: Comparative cross-sectional analysis of school based sleep intervention.

A series of sleep studies, including a national cross-sectional survey, a prospective cohort study, and a school based sleep intervention trial, were conducted in China from November 2005 through December 2009. The study profile is shown in **Figure 1**.

#### National cross-sectional sleep survey

Detailed information on subject enrollment was described previously [6], [19]. Briefly, a large national cross-sectional survey was undertaken in eight Chinese cities during November and December of 2005. 23,791 school-aged children were recruited from five/six grades (Primary school has five and six grades in Shanghai and other recruited cities, respectively) of 55 eligible primary schools in 39 districts. Of 23,791 children, 22,018 (92.5%) returned completed questionnaires. To eliminate the possible pubertal influences on the results of our study, 1240 children >12.00 years of age were excluded from the sample. The final sample consisted of 20,778 (boys 49.5% vs. girls 50.5%) school-aged children, with the mean age of 9.00 years (SD = 1.61; ranged from 5.08 to 12.00 years).

#### Prospective cohort study of sleep and school performance

Based on the national cross-sectional study, a sample of children in the first grade in Shanghai (n = 818, aged 6.80±0.31 years), who had complete sleep data, was enrolled as a prospective cohort subgroup (n = 818) to examine association of sleep with school performance. During December of 2009, 612 children aged 10.80±0.30 years (310 boys vs. 302 girls) participated in the five-year interval follow-up study and these children were included in the final analysis. Due to migration, transfer to another school, refusal of response, or missing data on sleep parameters or school performance at follow-up study, 206 (25.2%) children were dropped out during follow-up study. There was no significant difference in baseline characteristics (including age, gender, body mass index [BMI], family income, family structure, and parental educational levels) between children who participated in the follow-up study and those who did not.

#### School based sleep intervention trial

Out of a basic information screening in ten primary schools in Shanghai on September, 2007, six schools were selected as the eligible targets for the intervention trial according to three criteria: 1) no differences in sleep duration and daytime sleepiness between schools; 2) performing the same school schedule, including school starting time (7∶30am) and school finishing time (3∶30pm); 3) be similar in term of socioeconomic background and student educational achievements.

The intervention trial started from September, 2007 and continued to September, 2009. The intervention scheme employed a 3 groups (control, intervention1, and intervention 2) * 2 times (baseline, 2-year follow-up) model design. Each study group was randomly assigned 2 schools. After baseline screening and randomization, the school starting time delayed 30 minutes (from 7∶30am to 8∶00am) for intervention 1 and 60 minutes (from 7∶30am to 8∶30am) for intervention 2, meanwhile, control school kept the usual school schedule. During the intervention trial, to guarantee the standard academic and nonacademic activities, the school finishing time was correspondingly regulated, from 3∶30pm to 4∶00pm for intervention 1, from 3∶30pm to 4∶30pm for intervention 2, still 3∶30pm for control school.

Two rounds of cross-sectional sleep survey were executed during September, 2007 (just before the intervention trial) and September, 2009, separately. Given the trend of shorter sleep duration with increasing age, only students in the forth and fifth grades (Primary school in Shanghai has five grades) were specifically chosen as pre- and post-intervention comparative target analysis population.

During the first round survey, among 563 eligible children, 533 (94.6%) provided complete sleep data. With exclusion of 8 children >12.00 years to control the possible pubertal influences, the final sample consisted of 525 (boys 47.5% vs. girls 52.5%) children, with the mean age of 10.80 years (SD = 0.41; ranged from 9.00 to 11.92 years). For the second round survey, among 586 eligible children, 558 (95.2%) returned complete sleep data. With exclusion of 5 children >12.00, the final sample consisted of 553 (boys 50.8% vs. girls 49.2%), with the mean age of 10.81 years (SD = 0.33; ranged from 9.58 to 12.00 years).

### Measures

#### Sleep duration and daytime sleepiness

Sleep behaviors were assessed by a 36-item parents-administrated questionnaire, Children’s Sleep Habits Questionnaire (CSHQ), which was designed and developed to evaluate sleep characteristics from eight dimensions for pre-school and school-aged children, usually 4 to 12 years [24]. A Chinese version of the CSHQ was developed by translation, back translation, and psychometric property examination [25], which has been used previously with proven acceptable sensitivity and reliability, as described previously [6], [19].

The CSHQ included three items asking about bedtime, morning wake time, and daily total sleep duration during a “typical” recent week [24]. Based on sleep duration during weekdays and weekends, the mean sleep duration was calculated by averaging weekday and weekend sleep duration (weighted 5∶2 for weekday vs. weekend to account for the distribution of days in a week). So did the calculation for mean bedtime and mean morning wake time. In the present study, sleep duration <9 hours was defined as short sleep duration. This cut-off point was chosen because 9 hours is close to the 25^th^ centile of the distribution of total sleep duration in our national sleep study and reflects a clinically important degree of sleep restriction in the age group [26], [27]. Meanwhile, given the following reasons, sleep duration ≥10 hours per day was defined as sufficient sleep duration: 10 hours is close to the 75^th^ centile of the distribution of total sleep duration in the present study and was proposed as sleep recommendation for school-aged children [28]–[30].

In CSHQ, daytime sleepiness subscale included 8 items with respect to morning awakening difficulties and daytime tiredness. According to the CSHQ, the question was rated on a 3-point scale: “usually” if the problem occurred for 5–7 days per week, “sometimes” for 2–4 days per week, and “rarely” for 0–1 day per week. In the present study, frequent daytime sleepiness was defined as at least one item being “usually” and sometime daytime sleepiness was defined as at least one item being “sometimes”.

#### School performance

The teacher school achievement form (TSAF) was used to evaluate children’s school performance by their responsible teacher. TSAF was developed based on literature review [31]–[33], qualitative research, pilot studies, and reliability assessment. The final version of the questionnaire was conceptually grouped into 4 subscales: (1) attention and concentration (2 items); (2) interest and motivation (4 items); (3) academic achievement (5 items); and (4) school relationship (2 items). Each response was ranged by a five-point Likert scale: 1 for “Excellent”, 2 for “good”, 3 for “medium”, 4 for “not poor”, and 5 for “poor”. Upon the scoring system, the higher the score was, the more impaired the children’s school performance was.

(1) Subscale of attention and concentration: To assess the degree of paying attention to and focusing concentration on study in class or during doing homework.

(2) Subscale of interest and motivation: Four items were used to evaluate children’s interest and motivation toward study. For example, Can he/she do his/her homework on his/her own accord? Can he/she prepare lessons before class on his/her own accord? Do he/sh have learning strategy to promote his/her learning achievement? and Can he/sh take the initiative to overcome during learning?

(3) Subscale of academic achievement: This part included items with regard to achievement in listening and speaking, reading, writing, mathematical calculating, and logical analysis.

(4) Subscale of school relationship: Social relationship in school was investigated by two items: Can he/she do good job in teamwork? Can he/she get along well with peers and teachers?

The psychometric properties of the TSAF was found to be quite good (Cronbach’s α for the internal consistency is 0.83 for the overall questionnaire and ranges from 0.62 to 0.86 for subscales; intraclass correlation coefficients for the test–retest reliability is 0.85 for the overall questionnaire and ranges from 0.64 to 0.88 for subscales).

#### Demographic and Socioeconomic characteristics

Demographic variables included children’s gender, age, and ethnicity.

Family structure and socioeconomic variables included living area (urban area, suburban/rural area), family structure (single parent family, large family [the family with family members of grandparents, parents, and child], and nuclear family), parents’ educational levels (illiteracy, elementary or middle school, high school, college or university, and above university), and family income (<800, 800–2500, and ≥2500 Renminbi [RMB][yuan]/person/month).

Children’s height and weight were measured following the standard protocols by trained health workers and, then, were recorded in the questionnaire. Subjects were required to wear light clothes and stand straight, barefoot and at ease when being measured. The stadiometer and weight scale were both checked for accuracy by health worker before measurement. The same model and brand of stadiometer and weight scale were used for all children. Height was measured to the nearest 0.1 cm and weight was measured to the nearest 0.1 kg. Based on the parameters of height and weight, the body mass index (BMI) of the children was calculated: weight (kg)/height (m)^2^.

### Statistical Analysis

Univariate summary statistics and distributional plots were examined for all variables. Sleep parameters (bedtime, wake time, and sleep duration) were normally distributed. Subscale school performance scores were positively skewed and a logarithmic transformation was used to normalize the data for subsequent statistical analyses. Statistical descriptions were made by use of the mean, standard deviation for continuous variables, and percentage for categorical variables. Independent-sample *t* test, paired *t* test, one-way ANOVA, and Chi-square test were used to compare differences between groups where appropriate.

Penalized splines in generalized additive models were used to examine the linearity of relationships between sleep duration with school performance. Generalized cross validation was used to automatically select the degree of smoothing for spines. Generalized Linear Regression models were used to estimate the strength of the association of sleep duration and daytime sleepiness with school performance. Because no gender difference were observed with regard to sleep and school performance (all p for gender*sleep duration interaction >0.05, all p for gender*daytime sleepiness interaction >0.05), boys and girls were combined together in the final analyses.

All analyses were performed using the Statistical Analysis System (SAS) for Windows, version 9.2 (SAS Institute, Cary. NC), Statistical Package for Social Sciences (SPSS) for Windows, version 12.5 (SPSS Inc, Chicago**,** IL, USA), and R version 2.12.1 (The R Foundation for Statistical Computer, www.r-project.org). In the presentation of the results, the statistical significance was set at p value <0.05 (two tailed).

## Results

### Part One: Sleep Habits, Short Sleep Duration, and Daytime Sleepiness in School-aged Children by a National Cross-sectional Study

Sleep habits, including bedtime, wake time, and sleep duration, as well as prevalence of sufficient sleep, short sleep duration, and daytime sleepiness were summarized in **Table 1**. The mean sleep duration was 9.35±0.77 hours. 22.34% of children got sufficient sleep (≥10 hours per day). The prevalence of short sleep duration (<9 hours per day) and daytime sleepiness was shown to be 37.96% and 64.44% (sometimes 37.50% vs. frequently 26.94%), respectively. Most of sleep behaviors showed age, gender, ethnicity, district, and socio-economic differences. For example, compared to suburban peers, children in urban area slept less and showed higher prevalence of daytime sleepiness. Family income and parents’ educational levels were statistically related to almost all sleep behaviors, where it was demonstrated that higher family income and higher parents’ educational levels linked to shorter sleep duration.

**Table 1:** The association of sleep parameters with demographic and socioeconomic characteristics in the cross-sectional study (n = 20778).

	Sleep habits and prevalence of short sleep duration and daytime sleepiness								
	Bedtime	Wake time	SD	Sufficient SD	Short SD	Daytime sleepiness			
						No/rarely	Sometimes	Frequently	
	*mean±SD*	*mean±SD*	*mean±SD*	*n, %*	*n, %*	*n, %*	*n, %*	*n, %*	
Demographic characteristics									
Age (years)									
5–6 (2672, 12.9%)	21∶06±0.69	6∶59±0.51	9.56±0.76	891, 33.35	741, 27.73	947, 35.44	991, 37.09	734, 27.47	
7∼ (3802, 18.3%)	21∶13±0.68	7∶00±0.50	9.47±0.75	1050, 27.62	1189, 31.27	1320, 34.72	1494, 39.30	988, 25.99	
8∼ (3839, 18.5%)	21∶19±0.70	6∶59±0.49	9.34±0.74	842, 21.93	1454, 37.87	1391, 36.23	1385, 36.08	1063, 27.69	
9∼ (3798, 18.3%)	21∶25±0.71	7∶00±0.53	9.26±0.75	702, 18.48	1587, 41.79	1318, 34.70	1419, 37.36	1061, 27.94	
10∼ (3724, 17.9%)	21∶28±0.71	7∶02±0.52	9.23±0.76	600, 16.11	1634, 43.88	1337, 35.90	1385, 37.19	1002, 26.91	
≥11 (2943, 14.2%)	21∶35±0.77	7∶02±0.53	9.24±0.78	557, 16.93	1283, 43.59	1076, 36.56	1118, 37.99	749, 25.45	
*F/*χ^2^ value	185.46*** ^a^	18.18*** ^a^	100.62*** ^a^	383.53*** ^b^	309.54*** ^b^	14.94^b^			
Gender									
Boys (10227, 49.5%)	21∶20±0.73	7∶00±0.52	9.33±0.77	2239, 21.89	3993, 39.04	3628, 35.47	3845, 37.60		2754, 26.93
Girls (10445, 50.5%)	21∶22±0.72	7∶02±0.51	9.36±0.76	2378, 22.77	3846, 36.82	3721, 35.62	3909, 37.42		2815, 26.95
*t/*χ^2^ value	12.56*** ^c^	12.59*** ^c^	11.73*** ^c^	2.27^b^	10.84** ^b^	0.07^b^			
Ethnicity									
Han ethnic group (19604, 94.9%)	21∶22±0.72	7∶06±0.51	9.35±0.76	4392, 22.40	7416, 37.83	6994, 35.68	7342, 37.45		5268, 26.87
Minority ethnic group (1150, 5.1%)	21∶14±0.76	6∶55±0.47	9.33±0.78	227, 21.62	413, 39.33	353, 33.62	399, 38.00		298, 28.38
*t/*χ^2^ value	26.92*** ^c^	30.38*** ^c^	0.37 c	0.35^b^	0.96^b^	2.11^b^			
District									
Urban area (15866, 76.4%)	21∶22±0.54	6∶54±0.40	9.24±0.64	3786, 23.86	6064, 38.22	5833, 36.77	6340, 39.96		3692, 23.27
Suburban area (4888, 23.6%)	21∶17±0.64	7∶02±0.44	9.32±0.74	1154, 23.61	1655, 33.85	1899, 38.85	1899, 38.85		1090, 22.31
*t/*χ^2^ value	25.05*** ^c^	24.99*** ^c^	5.19** ^c^	0.04^b^	29.9*** ^b^	5.50** ^b^			
Family structure and socioeconomic status									
Family income (n, %)									
<800 (3956, 19.3%)	21∶08±0.74	6∶55±0.53	9.39±0.84	1030, 26.04	1328, 36.55	1413, 35.72	1387, 35.06		1156, 29.22
800–2500 (11612, 56.6%)	21∶20±0.71	7∶00±0.50	9.36±0.76	2661, 22.92	3326, 37.54	4174, 35.95	4397, 37.87		3041, 26.19
≥2500 (4966, 24.2%)	21∶34±0.69	7∶060.49	9.28±0.71	901, 18.14	1211, 39.95	1720, 34.64	1914, 38.54		1332, 26.82
*F/*χ^2^ value	397.51*** ^a^	154.16*** ^a^	27.26*** ^a^	83.72** ^b^	12.57** ^b^	19.94*** ^b^			
Family structure (n, %)									
Single parent family (1103, 5.3%)	21∶12±0.79	6∶57±0.71	9.33±0.72	266, 24.12	445, 40.34	393, 35.63	387, 35.09		323, 29.28
Large family (6565, 31.7%)	21∶21±0.56	7∶12±0.52	9.35±0.51	1498, 22.82	2447, 37.27	2346, 35.73	2526, 38.48		1693, 25.79
Nuclear family (13014, 62.9%)	21∶22±0.83	7∶06±0.76	9.34±0.76	2860, 21.98	4954, 38.07	4616, 35.47	4842, 37.21		3556, 27.32
*F/*χ^2^ value	25.64*** ^a^	7.17*** ^a^	0.50^a^	3.86^b^	4.04^b^	9.97* ^b^			
Mother’s educational levels									
Middle school and below (5752, 28.2%)	21∶09±0.76	6∶56±0.54	9.40±0.86	1624, 26.54	2293, 37.47	2247, 36.72	2255, 36.85		1617, 26.54
High school (6825, 33.4%)	21∶23±0.72	7∶01±0.51	9.34±0.76	1545, 22.64	2623, 38.43	2600, 38.10	2488, 36.45		1737, 25.45
College and above (7834, 38.4%)	21∶29±0.67	7∶04±0.48	9.31±0.69	1473, 18.80	2972, 37.94	2542, 32.45	3049, 38.92		2243, 28.63
*F/*χ^2^ value	379.83*** ^a^	104.45*** ^a^	24.52*** ^a^	119.07*** ^b^	11.25** ^b^	57.42*** ^b^			
Father’s educational levels									
Middle school and below (4940, 23.9%)	21∶07±0.77	6∶54±0.54	9.41±0.84	1373, 27.16	1876, 37.10	1819, 35.98	1863, 36.85		1374, 27.18
High school (7075, 34.2%)	21∶22±0.72	7∶04±0.52	9.34±0.78	1600, 22.61	2705, 38.23	2633, 37.22	2603, 36.79		1839, 25.99
College and above (8647, 41.8%)	21∶28±0.66	7∶04±0.48	9.31±0.70	1669, 19.30	3307, 38.24	2937, 33.97	3326, 38.46		2384, 27.57
*F/*χ^2^ value	437.90*** ^a^	141.49*** ^a^	25.99*** ^a^	113.91*** ^b^	2.09^b^	19.28*** ^b^			

SD: sleep duration; Family income was expressed in RMB(yuan)/person/month.

a Analysis of Variance, ANOVA.

b Chi-square Test.

c Independent-samples t test.

* *p*<0.05.

** *p*<0.01.

*** *p*<0.001.

### Part Two: The Association of Sleep Duration and Daytime Sleepiness with School Performance by a Five-year Longitudinal Cohort Study

#### Sleep parameters and sociodemographic characteristics of the sample

**Table 2** summarized sleep and sociodemographic outlines of the cohort study sample at baseline and follow-up surveys. All sleep parameters showed significant difference between baseline and follow-up, with the tendency of later bedtime and wake time, shorter sleep duration, lower prevalence of sufficient sleep, higher prevalence of short sleep duration, and higher level of daytime sleepiness at follow-up.

**Table 2:** The sample characteristics in the longitudinal study, 2005–2009 (n = 612, mean±SD unless indicated).

	Boys (n = 310)			Girls (n = 302)		
	Baseline	Follow-up	*t/*/χ^2^	Baseline	Follow-up	*t/*/χ^2^
Age (years)	6.80±0.31	10.80±0.30	564.27***a	6.79±0.31	10.80±0.30	608.74*** ^a^
BMI (kg/m^2^)	17.09±4.06	19.05±3.68	3.90***a	16.55±3.69	17.67±3.10	6.06*** ^a^
Family income, n (%)			4.67^b^			2.68^b^
Low (<800)	11 (3.59)	18 (5.96)		9 (3.02)	8 (2.77)	
Medium (800–2500)	143 (46.73)	118 (39.07)		142 (47.65)	119 (41.18)	
High (>2500)	152 (49.67)	166 (54.97)		149 (49.33)	162 (56.06)	
Family structure, (n, %)			0.34^b^			2.81^b^
Single parent family	10 (3.26)	12 (3.97)		6 (2.01)	8 (2.77)	
Large family	125 (40.72)	118 (39.07)		112 (37.46)	119 (41.18)	
Nuclear family	172 (56.02)	172 (56.95)		181 (60.54)	162 (56.06)	
Mother’s educational levels, n (%)			2.14^b^			3.62^b^
Middle school and below	83 (27.04)	79 (25.73)		72 (24.08)	64 (21.40)	
High school	87 (28.34)	74 (24.10)		89 (29.77)	74 (24.75)	
College and above	137 (44.63)	154 (50.15)		138 (46.15)	161 (53.85)	
Father’s educational levels, n (%)			0.49^b^			1.12^b^
Middle school and below	62 (20.20)	62 (20.85)		64 (21.40)	55 (18.39)	
High school	98 (31.92)	92 (29.32)		97 (32.44)	96 (31.77)	
College and above	147 (47.88)	153 (49.84)		138 (46.15)	149 (49.83)	
Bedtime	20∶46±0.53	21∶22±0.64	15.60*** ^a^	20∶45±0.52	21∶17±0.55	16.31*** ^a^
Wake time	6∶25±0.35	6∶36±0.35	7.68*** ^a^	6∶25±0.35	6∶35±0.40	8.30*** ^a^
Sleep duration	9.66±0.55	9.00±0.55	−14.70*** ^a^	9.67±0.50	9.07±0.60	−16.42*** ^a^
Sufficient SD, n (%)	103 (33.55)	16 (5.21)	78.90*** ^b^	98 (32.78)	24 (8.03)	56.39*** ^b^
Short SD, n (%)	23 (7.49)	137 (48.97)	106.90*** ^b^	24 (8.03)	102 (34.11)	61.18*** ^b^
Daytime sleepiness, n (%)			10.11** ^b^			10.67** ^b^
No/rarely	149 (48.53)	111 (36.16)		140 (46.82)	101 (33.78)	
Sometimes	91 (29.64)	120 (39.09)		99 (33.11)	120 (40.13)	
Frequently	67 (21.82)	76 (24.76)		60 (20.07)	78 (26.09)	

SD: sleep duration; Family income was expressed in RMB(yuan)/person/month.

a Paired t test.

b Chi-square Test.

** *p*<0.01.

*** *p*<0.001.

#### School performance at follow-up

Scores of each school performance subscale were positively skewed distributed. Median (P_25_–P_75_) was demonstrated as 4.0 (2.0–6.0) for subscale of attention, 8.0 (6.0–11.0) for subscale of learning motivation, 11.0 (9.0–15.0) for subscale of academic achievement, and 5.0 (3.0–6.0) for subscale of school relationship. After log-normalization transformation, the mean (SD) was shown as 1.35±0.46 (boys 1.47±0.44 vs. girls 1.23±0.44; *p*<0.001) for attention, 2.06±0.41 (boys 2.16±0.41 vs. girls 1.95±0.39; *p*<0.001) for learning motivation, 2.37±0.39 (boys 2.42±0.40 vs. girls 2.33±0.38; *p* = 0.003) for academic achievement, and 1.56±0.39 (boys 1.63±0.40 vs. girls 1.49±0.36; *p*<0.001) for school relationship.

#### Sleep duration and daytime sleepiness with school performance

**Figure 2:**
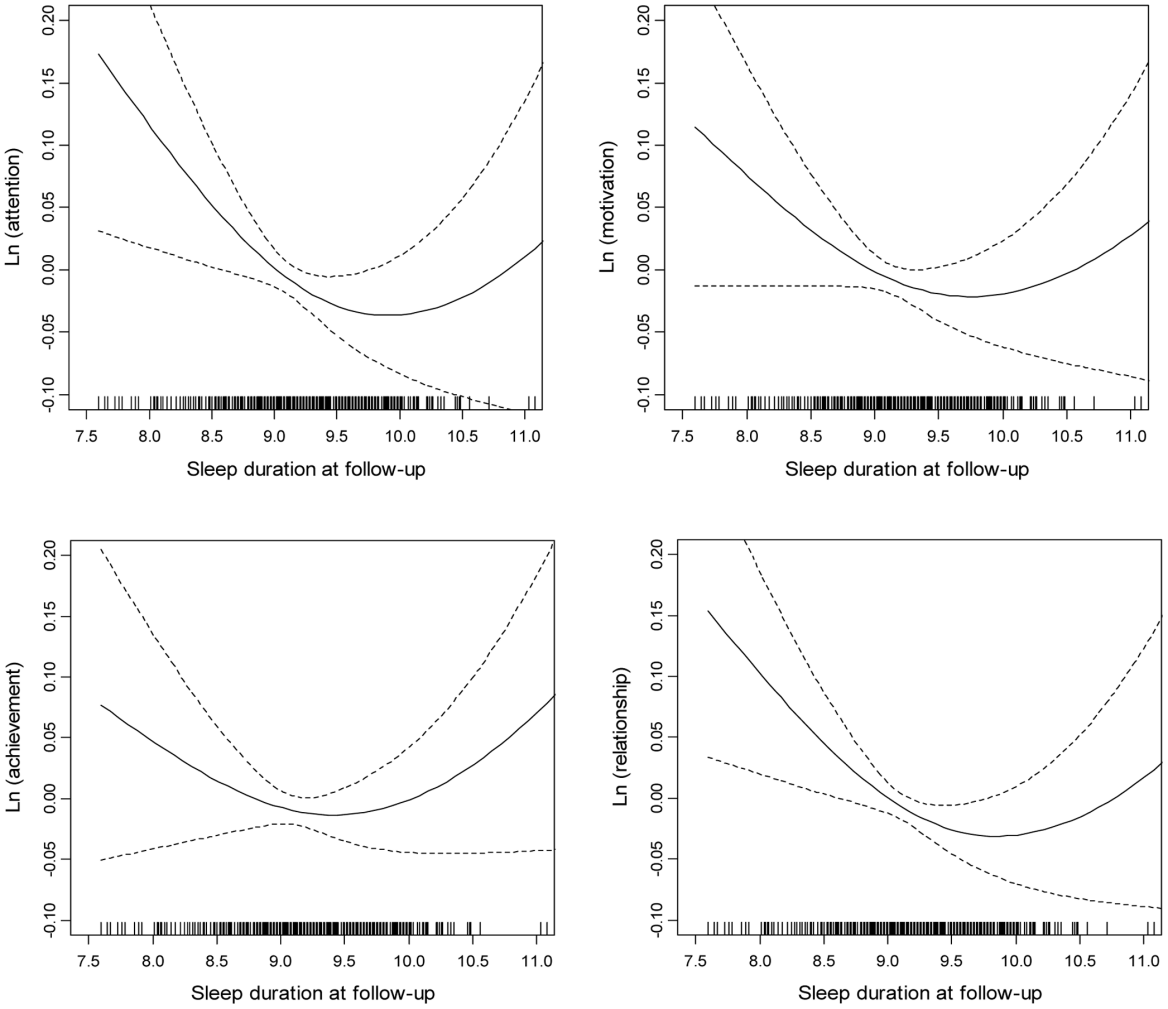
The associations between sleep duration and school performance at follow-up. The higher the score was, the more impaired the school performance was. Children’s gender, age, BMI, family income, family structure, and parents’ educational levels at follow-up were controlled.

**Potential nonlinear association of sleep duration with school performance by GAM models.** Using penalized spline of sleep duration, we observed a nonlinear association of sleep duration with school performance (*p* = 0.04) (as shown in **Figure 2**). The very similar nonlinear associations of sleep duration at baseline with school performance were also observed (not shown). Because of the nonlinear associations of sleep duration with school performance, the regression models were fitted with sleep duration as categorical variable (<9 hours and ≥10 hours compared with the middle 9–10 hours).

#### Baseline sleep duration and daytime sleepiness with follow-up school performance

After adjusted for age, gender, BMI, family income, family structure, and parents’ educational levels, the multivariate regression models revealed that the odds of impaired academic achievement in children with frequent daytime sleepiness was estimated to be 1.14 (*p* = 0.030) times greater than in children who didn’t have or rarely had daytime sleepiness. No significant association was observed between baseline sleep duration and follow-up school performance (**Table 3**).

**Table 3:** The Associations of sleep duration and daytime sleepiness, at baseline and follow-up, with school performance at follow-up in the longitudinal cohort study, 2005–2009 (n = 612).

	School performance at follow-up (outcome variables, as categorical variables)							
	AttentionHighest Quantile vs. other		MotivationHighest Quantile vs. other		AchievementsHighest Quantile vs. other		RelationshipHighest Quantile vs. other	
	OR (95% CI)	*P* value	OR (95% CI)	*P* value	OR (95% CI)	*P* value	OR (95% CI)	*P* value
**Baseline**								
Sleep duration (hours)								
<9	0.98 (0.83–1.17)	0.823	1.10 (0.92–1.30)	0.300	1.10 (0.92–1.31)	0.291	0.98 (0.82–1.17)	0.830
9–10	Ref		Ref		Ref		Ref	
≥10	1.03 (0.94–1.14)	0.511	1.04 (0.94–1.48)	0.418	1.03 (0.93–1.14)	0.536	1.04 (0.94–1.15)	0.457
Daytime sleepiness								
No/rarely	Ref		Ref		Ref		Ref	
Sometimes	0.94 (0.85–1.04)	0.209	0.95 (0.86–1.06)	0.361	1.04 (0.94–1.16)	0.428	0.99 (0.89–1.10)	0.827
Frequently	1.00 (0.89–1.12)	0.934	1.05 (1.00–1.18)	0.427	**1.14 (1.01**–**1.28)**	**0.030**	1.05 (0.93–1.18)	0.443
**Follow-up**								
Sleep duration (hours)								
<9	1.05 (0.96–1.15)	0.297	1.05 (0.96–1.15)	0.303	**1.20 (1.02**–**1.46)**	**0.049**	1.05 (0.95–1.15)	0.363
9–10	Ref		Ref		Ref		Ref	
≥10	1.11 (0.92–1.35)	0.288	0.99 (0.81–1.20)	0.899	1.09 (0.93–1.23)	0.061	1.07 (0.88–1.31)	0.502
Daytime sleepiness								
No/rarely	Ref		Ref		Ref		Ref	
Sometimes	1.06 (0.96–1.17)	0.269	1.07 (0.97–1.19)	0.159	1.01 (0.91–1.11)	0.882	0.92 (0.83–1.02)	0.117
Frequently	**1.14 (1.02**–**1.28)**	**0.030**	**1.11 (1.01**–**1.25)**	**0.015**	**1.18 (1.05**–**1.32)**	**0.005**	0.99 (0.88–1.12)	0.917

The higher the score was, the more impaired the school performance was.

OR, odds ratio; CI, confidence interval.

Generalized Linear Models controlled by children’s gender, age, BMI, family income, family structure, and parents’ educational levels.

#### Follow-up sleep duration and daytime sleepiness with follow-up school performance

At follow-up, the odds of impaired attention, learning motivation, and academic achievement in children with frequent daytime sleepiness was estimated to be 1.14 (*p* = 0.030), 1.11 (*p* = 0.015), and 1.18 (*p* = 0.005) times greater than in children who didn’t have or rarely had daytime sleepiness. Although sleep duration at baseline was not a significant factor influencing school performance at follow-up, sleep duration at follow-up played an important role in school performance, where it was shown that those children with sleep duration<9 hours had an increased risk for poor academic achievement (OR = 1.20, *p* = 0.049) (**Table 3**).

### Part Three: The Intervention Evaluation of Delaying School Start Time on Children’s Sleep by a Comparative Cross-sectional Study

#### Sleep parameters at baseline by control vs. intervention groups

**Table 4** summarizes sleep and sociodemographic characteristics of the intervention trial. Sleep parameters were very similar between control and intervention groups prior to intervention.

**Table 4:** The sample characteristics by groups at baseline (2007) and follow-up (2009) in the intervention trial (mean±SD unless indicated).

	The baseline survey (n = 525)					Post-intervention survey (n = 553)			
	Control(n = 158)	Intervention1(n = 215)	Intervention2(n = 152)	*F*/χ^2^		Control(n = 192)	Intervention1(n = 202)	Intervention2(n = 159)	*F*/χ^2^
**Socio-demographic characteristics**									
Age (years)	10.82±0.62	10.84±0.39	10.81±0.35	0.28^a^		10.81±0.45	10.82±0.36	10.81±0.42	1.23^a^
BMI (kg/m2)	17.46±2.99	17.69±3.33	18.36±3.98	2.47^a^		17.53±2.99	17.61±3.33	18.04±3.98	1.68^a^
Gender, n (%)				5.08^b^					4.82^b^
Boys	78 (49.68)	111 (51.86)	81 (53.29)			96 (50.73)	101 (51.00)	80 (50.62)	
Girls	79 (50.32)	104 (48.14)	71 (46.71)			94 (49.27)	97 (49.00)	79 (49.38)	
Family income, n (%)				6.75** ^b^					7.81** ^b^
Low (<800)	17 (10.83)	34 (15.81)	16 (10.66)			18 (9.83)	29 (14.26)	19 (12.30)	
Medium (800–2500)	119 (75.80)	134 (62.33)	82 (54.08)			140 (74.77)	121 (60.05)	84 (54.44)	
High (>2500)	21 (13.38)	47 (21.86)	54 (35.26)			29 (15.40)	47 (23.69)	52 (33.26)	
Family structure (n, %)				0.31^b^					0.26^b^
Single parent family	7 (4.49)	11 (5.12)	6 (3.97)			10 (5.52)	10 (5.08)	7 (4.32)	
Large family	44 (28.21)	61 (28.37)	42 (27.81)			52 (27.21)	55 (27.32)	44 (27.81)	
Nuclear family	105 (67.31)	143 (66.51)	103 (68.21)			129 (67.27)	135 (67.60)	107 (67.87)	
Mother’s educational levels, n (%)				13.20** ^b^					9.20** ^b^
Middle school and below	31 (19.62)	39 (18.14)	11 (7.24)			34 (17.62)	36 (18.10)	17 (11.32)	
High school	34 (21.52)	82 (38.14)	42 (27.63)			41 (21.52)	66 (33.18)	45 (29.72)	
College and above	93 (58.86)	94 (43.72)	99 (65.13)			116 (60.86)	97 (48.72)	90 (58.96)	
Father’s educational levels, n (%)				15.07*** ^b^					14.86*** ^b^
Middle school and below	30 (18.99)	49 (22.79)	20 (13.29)			32 (16.99)	41 (20.89)	17 (10.83)	
High school	39 (24.68)	66 (30.70)	43 (28.29)			38 (20.28)	58 (30.49)	47 (28.82)	
College and above	89 (56.33)	100 (46.51)	89 (58.42)			119 (62.73)	92 (48.62)	92 (60.35)	
**Sleep parameters**									
Bedtime	21∶19±0.59	21∶20±0.52	21∶25±0.44	1.95^a^		21∶32±0.56	21∶34±0.67	21∶37±0.62	7.29* ^a^
Wake time	6∶54±0.38	6∶52±0.37	6∶42±0.34	3.03^a^		7∶01±0.39	7∶13±0.47	7∶16±0.41	42.23*** ^a^
Sleep duration	9.17±0.73	9.20±0.63	9.18±0.58	1.47^a^		9.01±0.67	9.46±0.67	9.56±0.67	51.82*** ^a^
Sufficient SD, n (%)	24 (15.19)	29 (13.28)	21 (13.95)	1.02^b^		22 (5.73)	32 (15.68)	26 (16.39)	13.71** ^b^
Short SD, n (%)	68 (43.04)	89 (41.26)	62 (40.47)	2.62^b^		170 (44.27)	47 (22.88)	19 (11.87)	23.04*** ^b^
Daytime sleepiness, n (%)				4.79^b^					16.10** ^b^
No/rarely	64 (40.70)	76 (35.12)	53 (35.03)			71 (37.08)	87 (42.11)	86 (54.00)	
Sometimes	56 (35.44)	82 (38.14)	58 (38.03)			50 (26.30)	62 (29.11)	34 (21.32)	
Frequently	38 (23.86)	57 (26.74)	41 (26.95)			71 (36.61)	59 (28.78)	39 (24.67)	

SD: sleep duration; Family income was expressed in RMB(yuan)/person/month.

a Analysis of Variance, ANOVA.

b Chi-square Test.

* *p*<0.05.

** *p*<0.01.

*** *p*<0.001.

**The comparison of sleep parameters between control and intervention groups at post-intervention survey. As shown in** **Table 5** **,** during post-intervention survey, as compared to control group, intervention groups demonstrated longer sleep duration and decreased daytime sleepiness and the positive change was stronger in intervention 2 than in intervention 1.

**Table 5:** The comparison of sleep parameters between control and intervention groups after intervention in the intervention trial (mean±SD unless indicated).

	Post-intervention survey			
	Control	Intervention 1	Intervention 2	*F*/χ^2^
Bedtime	21∶32±0.56	21∶34±0.67	21∶37±0.62	7.29* ^a^
Wake time	7∶01±0.39	7∶13±0.47	7∶16±0.41	42.23*** ^a^
Sleep duration	9.01±0.67	9.46±0.67	9.56±0.67	51.82*** ^a^
Sufficient SD, n (%)	22 (5.73)	32 (15.68)	26 (16.39)	13.71** ^b^
Short SD, n (%)	170 (44.27)	47 (22.88)	19 (11.87)	23.04*** ^b^
Daytime sleepiness,n (%)				16.10** ^b^
No/rarely	71 (37.08)	87 (42.11)	86 (54.00)	
Sometimes	50 (26.30)	62 (29.11)	34 (21.32)	
Frequently	71 (36.61)	59 (28.78)	39 (24.67)	

SD: sleep duration.

a Analysis of Variance, ANOVA.

b Chi-square Test.

* *p*<0.05.

** *p*<0.01.

*** *p*<0.001.

**The comparison of sleep parameters between pre- and post-intervention surveys.** **Table 6** shows comparison of sleep parameters between pre- and post-intervention by control and intervention groups. In the intervention group, sleep parameters improved after the intervention. Each intervention group saw an increase in sleep duration, and a decrease in daytime sleepiness. The change was stronger in intervention 2 group than in intervention 1 group. For the control group, an opposite change was observed: a decrease in sleep duration and an up-regulation of daytime sleepiness.

**Table 6:** The comparison of sleep parameters between pre- (survey 1) and post-intervention survey (survey 2) in the intervention trial (mean±SD unless indicated).

	Control group			Intervention 1 group			Intervention 2 group		
	Survey 1(n = 158)	Survey 2(n = 192)	*t*/χ^2^	Survey 1(n = 215)	Survey 2(n = 202)	*t*/χ^2^	Survey 1(n = 152)	Survey 2(n = 159)	*t/*χ^2^
Bedtime	21∶19±0.59	21∶32±0.56	18.78*** ^a^	21∶20±0.52	21∶34±0.67	22.12*** a	21∶25±0.44	21∶37±0.62	20.02*** a
Wake time	6∶54±0.38	7∶01±0.39	4.58^a^	6∶52±0.37	7∶13±0.47	12.44*** ^a^	6∶42±0.34	7∶16±0.41	50.41*** ^a^
Sleep duration	9.17±0.73	9.01±0.67	−31.48*** ^a^	9.20±0.63	9.46±0.67	17.52*** ^a^	9.18±0.58	9.56±0.67	26.70*** ^a^
Sufficient SD, n (%)	24 (15.19)	22 (5.73)	12.90*** ^b^	29 (13.28)	32 (15.68)	8.63** ^b^	21 (13.95)	26 (16.39)	9.51** ^b^
Short SD, n (%)	68 (43.04)	170 (44.27)	0.07^b^	89 (41.26)	47 (22.88)	16.07***b	62 (40.47)	19 (11.87)	42.04***b
Daytime sleepiness, n (%)			13.68***b			13.92***b			25.17*** ^b^
No/rarely	64 (40.70)	71 (37.08)		76 (35.12)	87 (42.11)		53 (35.03)	86 (54.00)	
Sometimes	56 (35.44)	50 (26.30)		82 (38.14)	62 (29.11)		58 (38.03)	34 (21.32)	
Frequently	38 (23.86)	71 (36.61)		57 (26.74)	59 (28.78)		41 (26.95)	39 (24.67)	

SD: sleep duration.

a Independent-samples t test.

b Chi-square Test.

** *p*<0.01.

*** *p*<0.001.

## Discussion

### Main Findings and Significance

Using a series sleep design, the present study offers valuable insight into Chinese school-aged children’s sleep issues. The national epidemiological survey demonstrated that insufficient sleep and daytime sleepiness among Chinese school-aged children are very common. The longitudinal association of sleep with school performance showed that short sleep duration and, particularly, daytime sleepiness were positively associated with impaired attention, learning motivation, and academic achievement. Impairment of academic achievement was especially severe. The school-based sleep intervention emphasized the positive significance of optimal school schedule regulation for the improvement of children’s sleep. The significant contribution of our study lies in, for the first time, providing comprehensive knowledge of Chinese school-aged children’s sleep issues covering sleep pattern and its longitudinal association with school performance and, furthermore, exploring the application of sleep research in public policy.

#### Sleep duration and daytime sleepiness

Compared with their peers of other countries, such as the U.S, Belgium, and Switzerland, Chinese school-aged children slept approximately half an hour to nearly one hour less [27]–[33]. Guidelines for healthy sleep recommend that, over a 24 h period, school-aged children (6–12 years) require 10–11 hours of sleep [34]. By that criterion, only 22.34% of Chinese school-aged children get enough sleep. In addition, 9 hours of sleep duration reflects a clinically important degree of sleep restriction in the age group [26], [27]. By the cut-off, as high as 37.96% of Chinese school-aged children are going through clinically significant sleep restriction. Furthermore, daytime sleepiness, directly linked to impaired daytime functioning [7]–[10], was found to be 64.44% (sometimes 37.50% vs. frequently 26.94%), which was remarkably higher than their peers of other countries (15%−36.88%) [5], [35]–[37]. In addition, it should be pointed out that the daytime sleepiness is already highly prevalent from the earliest school age and it is maintained over the school years while sleep duration gradually declines. Previous study indicated that sleep need declines over the school age period from 11.4 hours at 5 years to 9.6 hours at 11 years [38]. In reality, because of the fixed school schedule, children are exposed to remarkable sleep loss at entry into primary school.

#### Sleep and school performance

In agreement with previous studies [22], [23], our study indicated that daytime sleepiness and in some cases short sleep duration are positively associated with the impairment of attention, learning motivation, and, in particular, academic achievement. The potential mechanism connecting sleep with academic achievement lies in the crosstalk between sleep and neurocognitive functioning, where it was proposed that advanced neurocognitive functioning, selective attention, generally abstract reasoning, goal directed behaviors, and creative processing, was characterized by an involvement of prefrontal cortex, which is known to be intensively sensitive to sleep and vulnerable to disrupted sleep [39], [40].

Although sleep duration and daytime sleepiness overlap to some extent, qualitative differences exist between them. The present study demonstrated that daytime sleepiness rather than sleep duration showed the most consistent and strongest relationship with school performance, suggesting that daytime sleepiness is more important to school performance. Basically, daytime sleepiness is the consequences of either poor sleep quality, insufficient sleep, or a combination of both. Therefore, daytime sleepiness could be considered as a reliable and significant clinical indicator for sleep screening in clinical practice. Moreover, our study further deepens the understanding that both early and concurrent daytime sleepiness are involved in the impairment of academic achievement. The finding emphasized the importance of sleep surveillance and intervention in the school setting. Finally and most importantly, our results provide a cautionary tale for the practice in Chinese society that children spend a lot of time on studies even with the sacrifice of sleep time [19], [21].

#### School-based sleep intervention evaluation

With greater awareness and understanding of the importance of sleep among children, increasing attention has been focused on the intervention strategy to promote childhood sleep health. Our previous study, along with other studies across different culture, reported that school schedule, particularly early school start time, contributed to sleep loss and daytime sleepiness [15], [16], [41]. Given the widespread epidemic of sleep loss in children, school schedule could be considered as a target for sleep intervention.

A similar intervention scheme was implemented in an American high school, which demonstrated that a 30-minute school start time delay was associated with an increase in sleep duration (45 minutes) and a decrease in the prevalence of daytime sleepiness (29.1%) [16]. However, as a pilot study in the field, several limitations, such as a limited setting (a high school), small sample size (n = 201), and lack of control group, restricted drawing general conclusions. Our intervention trial, adopting 3 (control, intervention1, and intervention 2) *2 (baseline, 2-year post-intervention follow-up) model design, was undertaken in an enlarged population of six primary schools. Taking into account the natural decrease of sleep duration during the intervention period, a moderate delay (30 minutes) of school start time significantly increased sleep duration (25.2 minutes) and decreased daytime sleepiness (8.99%). Delaying by another 30 minutes intensifies the benefits, however, the enlarged benefits are not very encouraging.

Although a positive effect was observed with respect to delaying school start time, it should be pointed out that only a minority of children got the recommended 10 or more hours of sleep and still approximately half of children continued to report daytime sleepiness after the change of the school start time. In addition, compared to the U.S pilot study among high school teenagers, our positive effect associated with delaying school start time was not impressive [16]. Further research is needed, where enriched and more comprehensive modification schemes covering changing school schedule, lightening academic burden, and intensifying sleep health education should be considered.

### Study Limitation

Several limitations should be taken into account in interpreting the findings of our results. First, parental reported data on children’s sleep parameters could increase the possibility of rater biases and inaccuracy. A more recent study, however, provided evidence that parental reports and actigraphic measures of sleep parameters among school-aged children were generally in agreement [42]. In addition, pubertal development assessment data was unavailable in our series study. Second, our sample size, especially in national cross-sectional study, is large, in which small effects are likely to become statistically significant. Therefore, it should be prudent to understand and explain our significant results. In addition, our longitudinal study only have two-point data. It is unavailable to analyze the tendency of sleep parameters to regress toward the mean. Third, this study did not have school performance measure at baseline and, therefore, could not examine its correlation between baseline and follow-up. IQ is an important confounder in association between sleep and school performance, however, our study included no measure of IQ. Forth, it is well known that mood regulation, behaviors, physical growth, and metabolic status are also involved in sleep associate implications [7]–[12]. A richer assay of health conditions could provide more information on the association of sleep with childhood well-being. Fifth, the present study specially focused on sleep duration and daytime sleepiness only, however, there may be other aspects of sleep, such as sleep disorders, sleep timing, and sleep hygiene, could partly explain the results that were found herein. Finally, our intervention trial is not a rigorous randomized control trial at the individual level, and as such, attribution of improved sleep to the regulation of school schedule alone should be prudent.

### Conclusion

In conclusion, our series sleep study gives insight into Chinese school-aged children’s sleep status, gives a reply to the concerns proposed in Chinese society where special emphasis was placed on studies at the cost of children’s sleep time, and provides valuable information on the application of sleep research in public policy.
